# Effect of Arm Positioning on Entrapment of Infraclavicular Nerve Block Catheter

**DOI:** 10.1155/2017/7196340

**Published:** 2017-02-28

**Authors:** Eric Kamenetsky, Rahul Reddy, Mark C. Kendall, Antoun Nader, Jessica J. Weeks

**Affiliations:** ^1^Department of Anesthesiology, Feinberg School of Medicine, Northwestern University, Chicago, IL, USA; ^2^Department of Anesthesiology, McGaw Medical Center, Northwestern University, Chicago, IL, USA

## Abstract

Continuous brachial plexus nerve block catheters are commonly inserted for postoperative analgesia after upper extremity surgery. Modifications of the insertion technique have been described to improve the safety of placing an infraclavicular brachial plexus catheter. Rarely, these catheters may become damaged or entrapped, complicating their removal. We describe a case of infraclavicular brachial plexus catheter entrapment related to differences in arm positioning during catheter placement and removal. Written authorization to obtain, use, and disclose information and images was obtained from the patient.

## 1. Introduction

Continuous brachial plexus nerve block catheters are commonly used to prolong postoperative analgesia after painful upper extremity procedures. Removal of these catheters is typically uncomplicated and often can be performed by the patient after hospital discharge. When peripheral nerve block catheters become damaged or entrapped, their removal can be challenging. Most reported cases of catheter entrapment are associated with epidural catheters. In these cases, it is recommended that the spine is flexed and continuous, gentle traction is placed on the catheter [1]. If these recommendations are applied to peripheral nerve block catheters, then, if resistance is met during removal, a patient's extremity should be positioned similar to when the catheter was inserted. We describe a unique case of infraclavicular brachial plexus catheter damage and entrapment related to differences in arm positioning during placement and removal of the catheter.

## 2. Case Description

A healthy 47-year-old male underwent left wrist radioscapholunate fusion for posttraumatic arthrosis. An infraclavicular brachial plexus nerve block was performed as the primary anesthetic, with an indwelling catheter placed for postoperative analgesia. After sterile preparation and draping of the left upper chest and positioning the left arm in an abducted and externally rotated position, a 2.5 cm linear array ultrasound transducer (13–6 MHz probe, SonoSite, S-Nerve™, Bothell, WA, USA) with sterile covering was used to visualize the infraclavicular brachial plexus. A medial infraclavicular approach was used as described by Bigeleisen and Wilson, with the needle puncture at the apex of the deltopectoral groove [2]. Prior to local anesthetic injection, a distal evoked motor response was obtained with a nerve stimulator, which disappeared at 0.4 mA. After performing a block through the needle with 30 mL 0.5% bupivacaine and 1 : 300,000 epinephrine, the 18 g × 4 cm continuous nerve block needle (Arrow International, Reading, PA, USA) was positioned with the tip between the posterior and medial cords of the brachial plexus. A 20 g × 60 cm continuous nerve block catheter (StimuCath®, Arrow International, Reading, PA, USA) was advanced 5 cm beyond the needle tip without resistance and secured with adhesive bandages with the 9 cm mark at the skin. A test dose of 5 mL 1.5% lidocaine with 1 : 200,000 epinephrine was administered through the catheter and an additional 10 mL of 0.5% bupivacaine was subsequently injected. Perineural spread near the posterior cord was confirmed with ultrasound during injection through the catheter.

**Figure 1:**
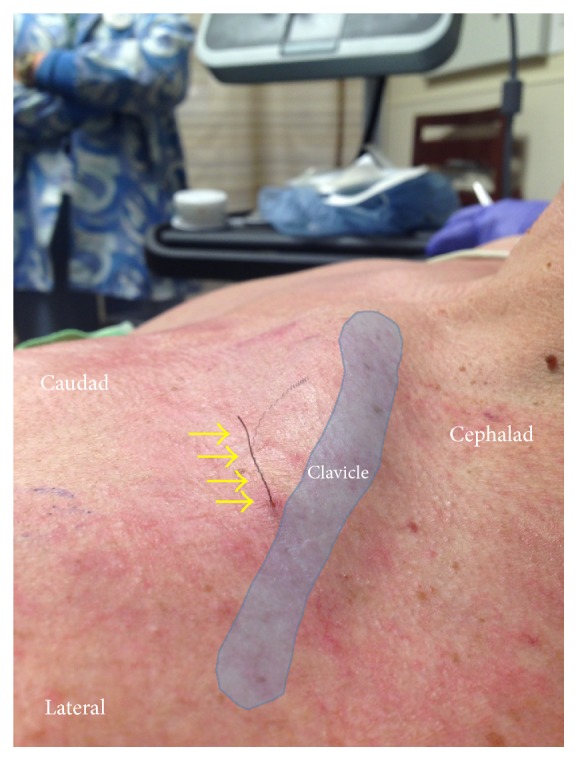
Wire fragment extending out of skin. During removal, the central stimulating wire and coil structure were noted to be fractured. Yellow arrows: wire fragment.

The patient successfully underwent the surgical procedure with the peripheral nerve block and intraoperative sedation. He was discharged home with the continuous nerve block catheter infusing 0.2% ropivacaine at 5 mL per hour, plus an additional patient-controlled dose of 2 mL per hour as needed. The patient reported excellent analgesia with 0/10 pain on postoperative days (POD) 1 and 2 and there were no signs of leakage or damage to the catheter. On POD 2, the entire 275 mL 0.2% ropivacaine infusion was completed. On POD 3, the patient went to the surgical office for a scheduled follow-up, at which time the surgeon attempted to remove the catheter with the arm adducted. Prior to removal, the patient reported no residual effects of the nerve block. The surgeon was able to remove the entire polyurethane catheter body without difficulty, but during removal, the central stimulating wire and coil structure were noted to be fractured, with approximately 3 cm remaining above the skin level (Figure 1). The patient reported a transient paresthesia down the left arm at the time of removal.

**Figure 2:**
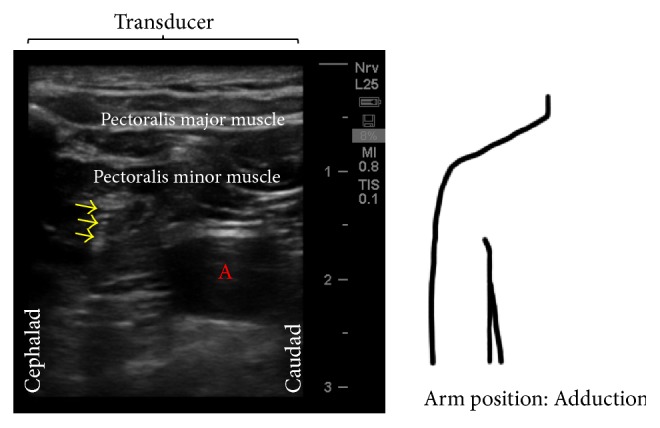
Infraclavicular neurovascular sonoanatomy with arm position adducted. The ultrasound image represents a transverse view with the top of the image displaying the ultrasound probe position. Arrows: needle shaft, A = axillary artery.

The patient was referred to the ambulatory surgery center for further evaluation by the anesthesiology team. On physical exam, the patient had normal strength and sensation in the left hand. He did experience sharp, severe, nonradiating pain near the clavicle upon abduction and external rotation of the arm, which resolved with adduction of the arm. Ultrasound evaluation did reveal the wire extending through the pectoralis major and minor muscles and coursing under the clavicle, immediately superficial to the brachial plexus. Since the arm was unable to be abducted, the infraclavicular neurovascular sonoanatomy was more challenging to interpret, as it appeared different than when the catheter was inserted (Figure 2).

**Figure 3:**
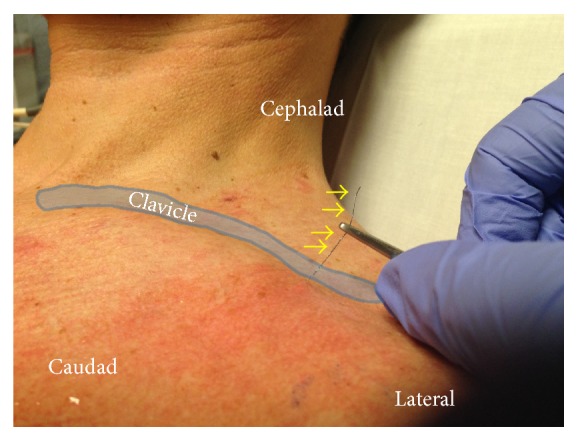
Removal of the fractured peripheral nerve catheter fragment. Traction was applied to the fragment while observing real-time removal with ultrasonography. Yellow arrows: wire fragment.

**Figure 4:**
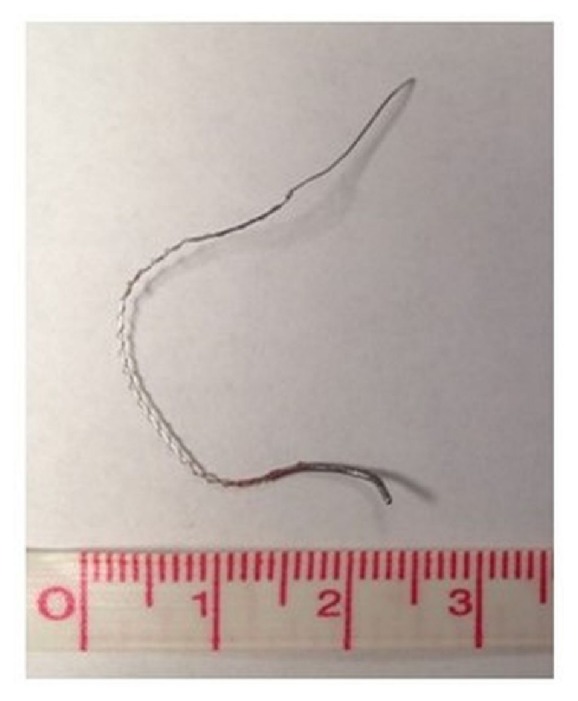
The stimulating wire and coil fragment following removal.

**Figure 5:**
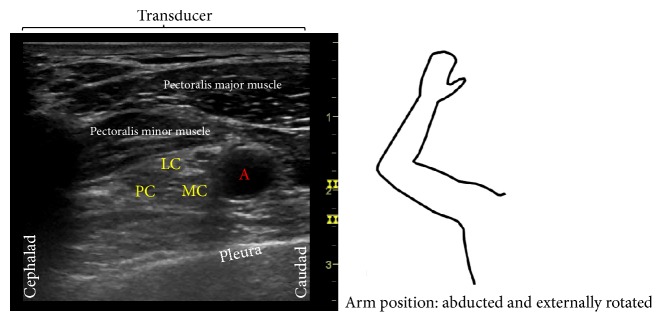
Follow-up ultrasound evaluation of the infraclavicular brachial plexus region with arm abducted and externally rotated. The ultrasound image represents a transverse view with the top of the image displaying the ultrasound probe position. A = axillary artery, LC = lateral cord, MC = medial cord, and PC = posterior cord.

A clamp was attached to the wire fragment and gentle traction was applied while observing the wire under real-time ultrasonography (Figure 3). During this process, there was no evidence of tension on the brachial plexus and the patient experienced no paresthesias. There was, however, significant resistance and local discomfort while initially withdrawing the wire. The wire was successfully removed with the tip intact (Figure 4). Of note, the wire fragment was visualized with ultrasound throughout the entire process and no retained fragments were seen after removal. Repeat physical examination showed full left shoulder range of motion without pain or paresthesias. No nerve block related complications were reported at a 2 week follow-up. During this follow-up visit, an ultrasound evaluation (GE Healthcare, Logiq™ P6, 11L probe, Chicago, IL, USA) revealed normal infraclavicular brachial plexus anatomy when the patients arm was abducted and externally rotated (Figure 5). No neurological adverse effects or complications were noted.

## 3. Discussion

Entrapped continuous peripheral nerve block catheters are most often due to a knotted, kinked, or damaged catheter [3–6]. Rarely, a catheter can become sheared, with a fragment remaining inside the patient. When a patient is symptomatic from a retained fragment, surgical exploration and removal may be required, particularly if there is no access to the catheter fragment above the skin level.

We describe a case of a damaged and entrapped infraclavicular nerve block catheter. The entire polyurethane catheter body was removed from the patient, but a fractured part of the stimulating wire and coil structure remained. Ultrasound was used to identify the course of the wire and confirm its position. Although the patient experienced local discomfort while withdrawing the wire fragment, bedside ultrasonography confirmed the fragment was intramuscular and was not causing tension on the brachial plexus or other important structures. The fragment was slowly withdrawn while being visualized in real-time with ultrasonography.

Previous studies described injecting saline through the catheter, to aid in unkinking the catheter or localizing its position in the patient [5]. This was not possible in our case, since the entire catheter body was removed. For the same reason, we were unable to apply electrical stimulation to the catheter to help determine the location of the fragment tip and proximity to the brachial plexus. We were also unable to visually assess the depth, as there were no depth markings. Excessive catheter advancement has been cited as a cause for difficult catheter removal [5]. The catheter in this case was threaded 5 cm beyond the needle tip without resistance and without moving the needle in the process. This was done to avoid leakage and accidental dislodgement of the catheter.

In this patient, the wire may have been entrapped in the costoclavicular space. The infraclavicular catheter was placed with the arm abducted and externally rotated, and the patient was unable to recreate this position due to severe shoulder discomfort. Positioning the arm in abduction and external rotation has been described by both Auyong et al. and Bigeleisen et al. as a means to bring the brachial plexus more superficial, distance the brachial plexus from the pleura, and move the clavicle out of the course of the needle [2, 7, 8]. Recent studies have described the clavicular motion that occurs with the arm abducted, as well as the changes in the costoclavicular space. Ludewig et al. showed that arm abduction to 110° in the coronal plane, similar to the arm position in our case, would result in a 10° increase in clavicular elevation angle, a 14° increase in posterior rotation, and 11° of clavicular retraction compared to a relaxed, neutral position [9]. LaBan et al. used computed tomographic (CT) imaging to evaluate the anatomic changes of the thoracic outlet in patients with clinical evidence of thoracic outlet syndrome. With the arm abducted to 90° and externally rotated, they found an average reduction in the costoclavicular space of 18.2 mm or a 55.6% reduction compared to the neutral position [10].

Several other studies have confirmed that there can be significant changes in clavicular position and space in the thoracic outlet with arm abduction. Although this arm positioning improved our ultrasound view of the brachial plexus during nerve block catheter placement, it may have complicated its removal. The patient was unable to recreate this position after initial catheter removal, due to severe shoulder pain with attempts at arm abduction. We hypothesize that initial catheter removal and subsequent removal of the wire fragment would have been straightforward and uncomplicated with the arm in an abducted position. The remaining fragment was likely entrapped in the costoclavicular space with the arm adducted causing pain with abduction.

Continuous nerve block catheter tip adhesion to the surrounding tissues may be another cause for catheter entrapment. Buckenmaier et al. studied catheter tip adhesion in a rat model using various tip designs provided by Arrow International. The rats randomized to the 19-gauge StimuCath catheter, similar to the catheter used in our case, required a nearly 20-fold greater mean force to remove the catheter tip after one week in a highly inflammatory intraperitoneal environment. In this study, the catheter remained in place for seven days, significantly longer than the three days in our case. Additionally, there was no evidence of adhesion formation on the fragment removed in our patient. A case series from Clendenen et al. described 5 cases of complications related to StimuCath catheter removal in which the polyurethane outer catheter cover became dislodged, while the metal core remained entrapped. In these cases, it is unclear if the catheter was damaged during placement or removal or if the StimuCath catheter tip design increases the propensity for entrapment compared to other catheters. Finally, another case reported by Duclas Jr. et al. describes a case of fibrous tissue adhesions complicating infraclavicular stimulating catheter removal. They suggest that a stimulating peripheral nerve catheter should be removed as soon as possible following completion of local anesthetic infusion to avoid the fibrous tissue growth that can occur. Whereas the usual practice at our institution is to instruct patients to remove the catheter at home on the day the local anesthetic is complete, the catheter in this case remained in situ for one additional day, potentially contributing to this complication.

An entrapped infraclavicular brachial plexus nerve block catheter fragment is a rare event and there are limited reports of similar events in the literature. Although previous authors have identified potential causes of nerve block catheter entrapment, our case highlights the importance of arm positioning during brachial plexus catheter removal and the challenges encountered if the arm cannot be repositioned. We also report the utility of ultrasound in this situation, specifically, how it can aid in locating a catheter fragment, confirming its proximity to the brachial plexus and visualizing its complete removal. Since this event, all patients are instructed to elevate their arm above their head prior to removal of the catheter, if their arm was abducted during catheter placement.
